# Human language evolution: a view from theoretical linguistics on how syntax and the lexicon first came into being

**DOI:** 10.1007/s10329-021-00891-0

**Published:** 2021-04-05

**Authors:** Haruka Fujita, Koji Fujita

**Affiliations:** Graduate School of Human and Environmental Studies, Kyoto University, Yoshida Nihonmatsu-cho, Sakyo-ku, Kyoto, 606-8501 Japan

**Keywords:** Language evolution, Merge, Lexicon, Motor control origin, Disintegration, Functional categories

## Abstract

Human language is a multi-componential function comprising several sub-functions each of which may have evolved in other species independently of language. Among them, two sub-functions, or modules, have been claimed to be truly unique to the humans, namely hierarchical syntax (known as “Merge” in linguistics) and the “lexicon.” This kind of species-specificity stands as a hindrance to our natural understanding of human language evolution. Here we challenge this issue and advance our hypotheses on how human syntax and lexicon may have evolved from pre-existing cognitive capacities in our ancestors and other species including but not limited to nonhuman primates. Specifically, we argue that Merge evolved from motor action planning, and that the human lexicon with the distinction between lexical and functional categories evolved from its predecessors found in animal cognition through a process we call “disintegration.” We build our arguments on recent developments in generative grammar but crucially depart from some of its core ideas by borrowing insights from other relevant disciplines. Most importantly, we maintain that every sub-function of human language keeps evolutionary continuity with other species’ cognitive capacities and reject a saltational emergence of language in favor of its gradual evolution. By doing so, we aim to offer a firm theoretical background on which a promising scenario of language evolution can be constructed.

## Introduction

Language is a uniquely human trait, and in the past this simple fact long stood as a conceptual and methodological barrier for a natural understanding of how this mental capacity first came into being only in our species. The barrier has been removed by the modular view of the human language faculty that it is not a monolithic trait but a multi-componential function integrating several autonomously working sub-functions each of which evolved in humans and other species independently of language. In linguistics, this modular view has been strongly advocated by generative grammar (Chomsky 1965, 1986 inter alia). While semantic and phonological modules have obvious evolutionary continuity with other species’ capacities,^1^ two modules have been claimed to be truly unique to the human species—the syntactic computational system (syntax) and the lexical system which provides inputs to the computation (the lexicon) (Hauser et al. 2002; Berwick and Chomsky 2016). This kind of species-specificity poses a serious problem if we are to understand language as something evolved from pre-existing capacities of human and nonhuman species. In this article, we tackle this problem and argue that the origins and evolution of human syntax and lexicon can receive a natural explanation by combining insights from theoretical linguistics with what has been known about human and nonhuman cognition.

## The basic architecture of human language

Roughly, human language is a system connecting sound and meaning via hierarchical syntactic structure. The property of structure dependence, that human language is crucially dependent on hierarchical structure rather than (or in addition to) linear structure (word order), is a distinctive feature of language not shared by other systems of animal communication (Everaert et al. 2015). To illustrate, (1a) has two different semantic interpretations and phonological realizations (prosody) depending on which of the two structures in (2) it has.

1. a. green tea cup
  b. modern Japanese dictionary

- will mean “a tea cup which is green,” and (2b) “a cup for green tea.” Likewise, (1b) is ambiguous between “a Japanese dictionary which is modern” and “a dictionary of modern Japanese.” Similarly, many sentences are structurally ambiguous.
- a. The dog thinks the cat fell again.
  b. The dog which barks often sleeps.

In (3a), the adverb *again* can be part of the matrix clause (“the dog thinks again”) or of the subordinate clause (“the cat fell again”). In (3b), *often* can be in the matrix clause (“the dog often sleeps”) or inside the relative clause (“the dog barks often”).

This property of structural ambiguity is a potential risk to efficient communication because it gives rise to the danger of misunderstandings between the speaker and the hearer. It follows that the evolution of hierarchical syntax is hard to explain if we focus on communication alone. There is a long-standing controversy among researchers, linguists and biologists alike, over the question of what the fundamental or original function of language is. While many agree that language is primarily for communication, there are also those who think otherwise. For the latter, hierarchical syntax is adaptive for forming complex thoughts by hierarchically combining concepts. Fitch (2019) also states: “language … includes recursive compositional machinery that allows us to flexibly combine basic concepts into complex, hierarchically structured thoughts.” To us, this seems to be a natural and promising view, as already illustrated by the examples (1)–(2) above, and to the extent that it is, it will be a serious mistake to try to derive human language only from animal communication in an evolutionary context. To say the least, communication is not the only function of language, and language alone is not enough for communication. See also Fujita (2016) for some discussion on the “fallacy of communication” and then on the “fallacy of thought.”

Noam Chomsky’s generative grammar (Chomsky 1965, 1986 inter alia) is an enterprise to study human language as our biological endowment. As such, it is intended as part of biolinguistics, biological studies of language, covering a wide range of related research fields not limited to linguistics (Boeckx and Grohmann 2013). Sometimes biolinguistics is even equated with generative grammar in the literature but that is an unwarranted view (see Martins and Boeckx (2016) for relevant discussion). We assume, however, that generative grammar provides a, not *the*, firm theoretical foundation for today’s biolinguistics. The basic tenet of generative grammar is that humans, and only humans, share the innate biological endowment for language—Universal Grammar (UG). Whether UG really exists and, if so, what it is precisely, remain to be seen, but since by definition UG constitutes our biologically determined capacity which emerged in the evolutionary history of humans, the issue of the origins and evolution of language is directly relevant to generative grammar.

**Fig. 1:**
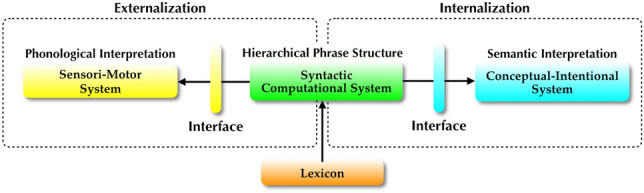
Basic architecture of the human language faculty. The lexicon provides inputs to the syntactic computational system (syntax), which combines these lexical items into a hierarchical phrase structure. This structure is then transferred to the two interpretive systems, the conceptual-intentional (CI) system for semantic interpretation and the sensorimotor (SM) system for phonological interpretation, including signs and other forms of surface realizations, via the two interfaces which roughly correspond to Logical Form (LF) and Phonetic Form (PF) in earlier versions of generative grammar. The syntax-CI connection is adaptive for internalization that takes place within an individual (thought, inference, planning, etc.), while the syntax-SM connection is for externalization (communication with other individuals)

Adopting the terminology of current version of generative grammar, known as the minimalist program or minimalism (Chomsky 1995 et seq.), the basic design architecture of human language can be shown as in Fig. 1.

An important hypothesis upheld in the minimalist program is that language (more precisely, the computational system) is optimally designed only for internalization, and externalization is a peripheral process (Berwick and Chomsky 2016). The hierarchical structure derived by the computational system is already enough for the purpose of semantic interpretation (in this sense, semantics is part of syntax), while externalization requires many language-specific adjustments, including conversion of this hierarchical structure to a linear structure and morphophonological realization of each element appearing in the structure.

This simple observation effectively illustrates that while internalization is universal, there is every kind of cross-linguistic variation with respect to externalization. In fact, linguistic diversity is found only in the domain of externalization and there is only one human language as far as internalization goes. In short, internalization is simple but externalization is complex, and the so-called cultural evolution of language, which should be distinguished from biological evolution even though the two are likely to be closely related to each other, is all a matter of how externalization is subject to change.

Turning back to Fig. 1, to the extent that language has such a modular design, the question of language evolution boils down to the evolution of these modules (sub-functions) and how they got combined into the complex system we know as language. The widely held view is that while the two interpretive systems have homologues or analogues in other species, such as concept formation of primates and vocal learning of songbirds, syntax and the lexicon may constitute truly species-specific capacities. Very famously, Hauser et al. (2002) advanced the hypothesis that recursion may be the only component of language which is unique to humans and the human language (faculty of language in the narrow sense; FLN). Recursion here refers to the syntactic computational system itself, not to be confused with clausal embedding or any other specific constructions. Generally speaking, a recursive function takes its own output as its input. To illustrate, in (2a) {tea, cup} is the output of the combinatorial computation working on *tea* and *cup*, and subsequently the same computation works on this output and *green*, forming {green, {tea, cup}}. The evolution of such an operation is key to our understanding of language evolution.

Not only that, we also need to explain how the computational atoms, the lexicon, evolved. Human lexical items are qualitatively different from animal signals as they represent concepts rather than directly refer to objects in the outside world. The vervet monkey’s alarm calls for different types of predators (Seyfarth et al. 1980), for instance, are better understood as an automatic response to the approaching danger instead of a signal standing for the abstract concept of a specific kind of predator itself, as human words do. Berwick and Chomsky (2016) discuss this property and state that human lexical items “pose deep mysteries,” their origin being “entirely obscure” (p.90). Below we present a possible scenario of the evolution of the human syntax and lexicon by bringing together the insights from theoretical linguistics, not limited to generative grammar, and those from other relevant fields including comparative cognitive psychology. For that purpose, let us first take a closer look at each of these systems.

## Merge: the generative engine of human language

As stated above, human language is dependent on hierarchical structure. How such a structure is generated has been one of the main topics throughout the history of generative grammar. In the past, highly language-specific rule systems and principles, like phrase structure rules, transformational rules, X-bar theory and Move α (the details of which need not concern us here), were proposed. These were not able to meet the evolvability condition; given the very short evolutionary time of language (presumably within 100–200 thousand years), there is simply no way for such complex knowledge to evolve in our species. In the minimalist program, all these rules and principles have been abandoned in favor of the most fundamental computational operation called “Merge” (Chomsky 1995 et seq.). Merge is the only generative engine of human language, and it is claimed to be the only new function necessary for language to evolve from the already existing capacities (precursors). This “Merge-only” view (“All you need is Merge”; Berwick 2011) provides an excellent conceptual starting point in our discussion of language evolution.

By definition, Merge is a recursive operation that takes two syntactic objects (lexical items or their sets already defined by Merge) and forms one unordered set.^2^

(4) Merge (α, β) → {α, β}

Merge is a unitary operation, and only for convenience sake two modes of applying Merge can be distinguished. Where α and β are initially external to each other (neither is part of the other), we have an instance of External Merge (EM). Where either α or β is included in the other, we have an instance of Internal Merge (IM).

(5)

Internal Merge was formerly known as Move [in (5b), β moves from its original position to the highest position], and is the source of the “displacement” property of human language such that an element receives its semantic interpretation in one position but is pronounced elsewhere.

(6) a. John was arrested (John).
  b. Mary wonders what John did (what).

In (6), *John* and *what* are each semantically interpreted in the object positions but then they are pronounced in the clause-initial positions. Animal communication systems do not show this displacement property. Merge is the root of many properties unique to language, including displacement as well as compositionality, recursiveness, and the hierarchical nature of linguistic structures. As such, the evolution of Merge is of principal importance to our understanding of human language evolution. Unfortunately, however, the evolution of Merge is seldom addressed seriously, except the occasionally made vague suggestion that a simple mutation causing the rewiring of the brain is responsible for it (Berwick and Chomsky 2016). Given that everything in biological evolution is continuous, it is only natural to assume that Merge evolved from some precursor instead of saltationally appearing from nowhere, and the question to be answered is what this precursor was.

It is interesting to note in this connection to observe that a Merge-like combinatorial operation can be observed in other species particularly in the motor action domain. Tool use is an exemplary case, where animals manipulate concrete objects hierarchically. The well-documented nut-cracking behavior of chimpanzees can be described using a tree-structure representation as in (7) (Matsuzawa 1996: 204).

(7)

The formal parallelism between motor action and linguistic structure is explored among others by Greenfield’s (1991, 1998) “action grammar” paradigm. Her proposed three strategies of combining objects (like nesting cups) can be directly translated into different modes of applying Merge. The simplest mode of Merge is just a combination of two linguistic objects, which we call Proto-Merge because this is the most primitive, non-recursive form of Merge which would have preceded recursive Merge in language evolution (Progovac 2015).

(8)

Proto-Merge corresponds to Greenfield’s Pairing strategy. When this Proto-Merge applies iteratively, like combining *green* with (8a), we have an instance of what we call Pot-Merge, after Greenfield’s Pot strategy.

(9)

The third mode of Merge, which we call Sub-Merge after Greenfield’s Subassembly strategy, can be illustrated by (10), where (8b) serves as a subassembled chunk to be next combined with *cup*.

(10)

Subassembly Strategy is the most complex way of combining objects and, with minor exceptions (Hayashi and Takeshita (2020); captive chimpanzees with intensive training, pointing to the relevance of this strategy to language), it is in principle unique to the humans (Conway and Christiansen 2001). Accordingly, Sub-Merge is the nuts and bolts of human language syntax. It must be utilized in deriving a simple sentence as in (11).

(11) a. The boy met a girl.
  b. [_*v*P_ [_NP_ the boy][_*v*’_ *v* [_VP_ met [_NP_ a girl]]]]
  c. 
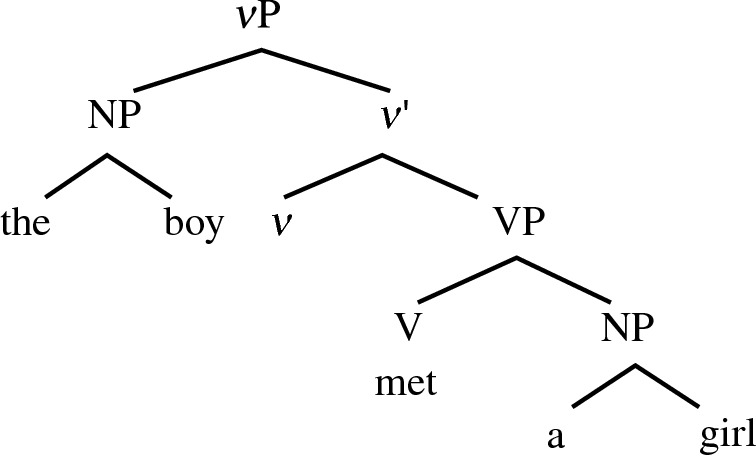

Using a simple X-bar notation, the VP structure contained in (11a) can be shown as in (11b) = (11c). It is fairly easy to see that without Sub-Merge the structure is not derivable at all.

**Fig. 2:**
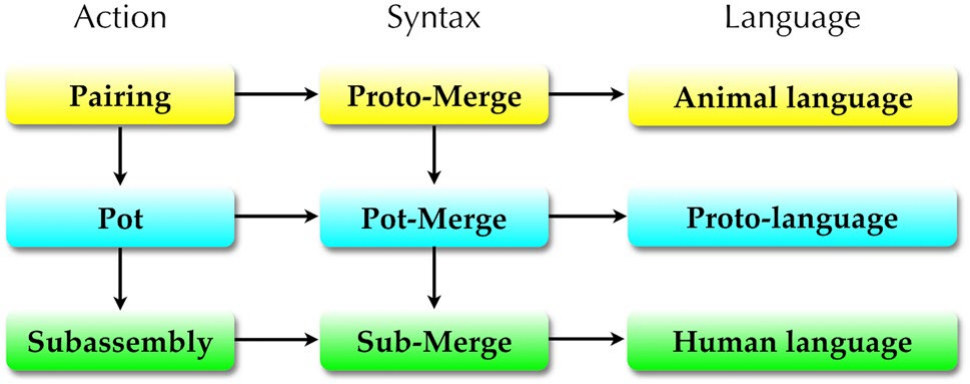
Motor control origin of Merge. Linguistic Merge evolved from action Merge (action grammar). Starting from the Pairing strategy of action Merge, motor action and linguistic syntax evolved into more and more complex forms. Proto-Merge characterizes animal communication systems (metaphorically, animal “language”), where at most only two (sets of) signals are combined (Miyagawa and Clarke 2019), as in the *pyow-hack* sequence of putty-nosed monkeys (Schlenker et al. 2016a) and the ABC-D call of the Japanese tit (Suzuki et al. 2018). Protolanguage had at best Pot-Merge, given the plausible assumption that it had only linear syntax (Jackendoff and Wittenberg 2016) and hierarchical syntax was yet to come. With the advent of Sub-Merge, exapted from Subassembly strategy of action Merge, human language with all its structural properties emerged

Observations like the above led one of the authors to propose the hypothesis of the Motor Control Origin of Merge (Fujita 2009, 2014, 2017a inter alia), according to which linguistic Merge evolved or was exapted from “action Merge” (Greenfield’s action grammar). Succinctly, this hypothesis can be shown as in Fig. 2.^3^

This scenario of how Merge may have evolved from an already existing motor action raises some new questions to be addressed. Two major questions are: (1) why only humans were able to extend motor action to linguistic computation, and (2) why only humans were able to go from Pot strategy (Pot-Merge) to Subassembly strategy (Sub-Merge). We have no definitive answers but here are some possible ways to proceed. Obviously, linguistic Merge requires ingredients to which it can apply, and to the extent that other species have no linguistic atoms (lexical items) there is no possibility that they have linguistic Merge. Only humans combine abstract concepts into complex structures because they have a process of “metaphorical extension,” by which we mean humans can handle concepts as manipulatable concrete objects (which is not to be confused with word meaning extension by metaphor). This is already illustrated by the examples (9) and (10) above, where the lexical concepts combined by Pot strategy (Pot-Merge) or Subassembly strategy (Sub-Merge) newly form distinct complex concepts. We further speculate that this extension became possible by externalizing those concepts in the form of words and other linguistic symbols. If on the right track, this consideration suggests that, contrary to the minimalist thinking, externalization is not a peripheral phenomenon in language evolution but is as important as internalization. Externalization feeds internalization to render the latter more elaborate, and there is a co-evolutionary relation between them (see Fujita 2020 for relevant discussion). Furthermore, the role of the lexicon in language evolution is immense here, and we are not allowed to leave it simply as a “deep mystery.” For more on this, see the section “The evolution of roots and functional morphemes.”

With respect to the move from Pot-Merge to Sub-Merge, an obvious factor is advanced working memory in humans. But it is interesting to observe here that Sub-Merge requires a kind of “multiple attention” which is not needed in Pot-Merge. By this we mean that paying attention to more than one target of operation is necessary in Sub-Merge but not in Pot-Merge. For instance, in (9), *cup* is constantly the only target, to which *tea* and *green* are “attracted” sequentially, but in (10), *tea* and *cup* function as two separate targets to which attention needs to be paid equally. A further speculation we might make is that this multiple attention results from human “self-domestication,” which plays a significant role in human evolution and human language evolution (Benítez-Burraco and Kempe 2018; Thomas and Kirby 2018). Domestication yields many physiological, morphological and cognitive changes collectively known as the “domestication syndrome” (Wilkins et al. 2014), among which are reduced fear of novel objects and enhanced curiosity. These will allow the animal to refrain from quickly responding to a stimulus and to consider different steps to take in a given situation. Multiple attention will result from such behavioral uniqueness. Humans are not the only self-domesticated species, and therefore multiple attention is not the only factor in the evolution of complex hierarchical syntax. The gist of this proposal is that the evolution of Merge may also be discussed in a sociocultural context (since domestication has an effect on prosociality, friendliness, tameness, etc.), a view completely missing in the minimalist approach.

## Generic Merge

In generative grammar, Merge is understood to be a uniquely human, language-specific capacity. More importantly, Merge is assumed to be the only component of Universal Grammar (UG) under minimalist theorizing which aims at maximally simplifying this specifically linguistic genetic endowment of the human species. This research strategy has methodological benefits for our understanding of language evolution because it minimizes what has to be explained in order for human language to be evolvable at all. The motor control origin of Merge just presented claims that Merge may be an exaptation of motor action planning shared not only by humans but by other species. Ironically, considerations like this raise the possibility that Merge may not be so species-specific or domain-specific as widely believed in generative grammar. It may be that linguistic Merge is one instance of domain-general combinatorial capacity (call this “generic Merge,” extending Boeckx’s (2008) terminology) becoming domain-specific in the process of evolution and/or development, in which case it will follow that there is actually no such thing as UG to be explained in terms of our biological evolution.

Similar views are not new at all. Hauser and Watumull (2017) propose the “Universal Generative Faculty (UGF)” as the domain-general generative engine shared by, e.g., language, mathematics, music and morality. Marcus (2006) already argued that “descent-with-modification modularity,” as opposed to “sui generis modularity,” is the right kind of modularity to understand the domain-specific nature of cognitive modules. Domain-specific properties of linguistic Merge will then be a result of this UGF interfacing with a particular domain of linguistic knowledge. For instance, by definition linguistic Merge forms an unordered set where only information on hierarchical relations is available. Action Merge, by contrast, is inherently ordered, its outputs being both linear and hierarchical. This difference may reflect the fact that abstract linguistic symbols, but not concrete objects, can be manipulated in an order-free way. Also, it is generally accepted in minimalist literature that Merge is a binary operation that always combines just two linguistic elements. Accordingly, linguistic structure is always binary-branching. This property may not be shared by other cognitive domains, say music. If so, the binarity of linguistic Merge may be a result of it operating in the linguistic computation. Specifically, outputs of linguistic Merge need to be subsequently linearized for externalization, and this linearization process involves converting hierarchical structure to sequential structure. Notice that binary-branching structure as in (12a) is easier to linearize than, say, ternary-branching structure as in (12b).

(12) a. [ A [ B C]]
  b. [ A B C]

In (12a), it is obvious that A is higher than B or C and therefore A comes first in linear order if, as seems natural, linearity reflects hierarchical relations (see Kayne (1994) for the relevant idea of Linear Correspondence Axiom). Assuming that the relative order of B and C is yet to be determined by some means, (12a) allows two different linearization patterns. In (12b), however, there is no highest element to be linearized first, and the structure allows as many as six different linearization patterns. The number of possible word order increases as there are more branching. Accordingly, binary branching is an optimal solution to the linearization problem.

**Fig. 3:**
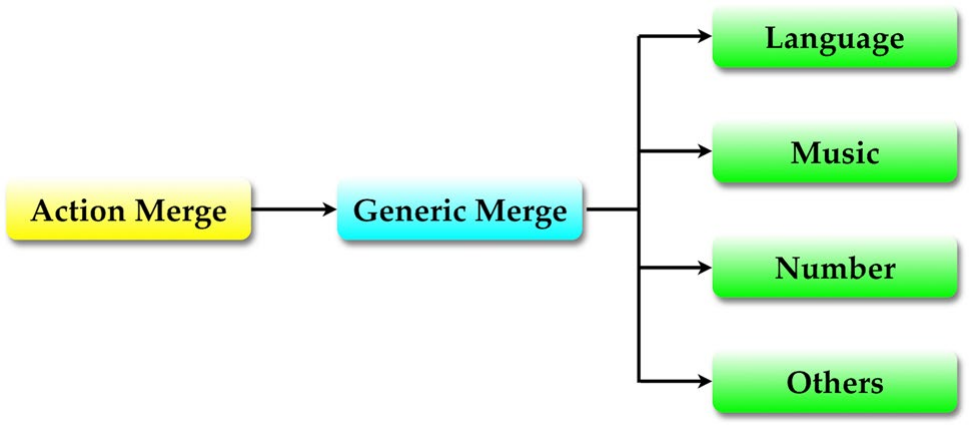
From action Merge evolved a domain-general combinatorial operation (generic Merge), and from the latter derive a variety of domain-specific operations including linguistic Merge. The formation of generic Merge corresponds to what Hauser (2009) refers to as “the release of recursion from its motor prison to other domains of thought.” A possibility is that only this generic Merge is our innate capacity, and domain-specific operations develop through ontogeny, much in line with Marcus’ (2006) descent-with-modification version of modularity. If the minimalist thinking is correct that UG contains only Merge and nothing else, it will follow that there is no UG

Turning back to the motor control origin of Merge, what evolved from motor action planning may be such a generic version of Merge, which in turn serves as a precursor to a variety of domain-specific combinatorial operations including linguistic Merge, as in Fig. 3.

If this view is correct on the whole, then Merge will lose its status as an explanandum in language evolution, and the evolution of the human lexicon, which provides inputs to Merge, becomes all the more important, instead. We now turn to this topic.

## The definition of the lexicon: based on Distributed Morphology

In generative grammar, there have been two contrasting standpoints regarding the relationship between the lexicon and syntax: lexicalism and anti-lexicalism. Lexicalism sets the lexicon as an independent generative module responsible for creating words, with syntactic structure being generated in conformity with the lexical information of those words. Anti-lexicalism maintains that both words and sentences are generated by syntax and their semantic-conceptual interpretation is determined by syntactic structure. Lexicalism has been the dominant framework throughout the history of generative grammar, but with the advent of the minimalist program, the possibility of anti-lexicalism has also attracted much attention as it allows us to investigate the direct relationship of syntax and the lexicon. For one thing, minimalism rejects many theoretical assumptions upon which lexicalism was built. Typically, the distinction between the base and transformational components was abandoned in favor of the single syntactic computational system (Merge).

Anti-lexicalism is a promising approach from the perspective of theoretical linguistics in that it can explain the immediate relation between syntax and morphology (as captured by Baker’s “Mirror Principle” (Baker 1985), for instance) in addition to significantly reducing lexical entries. More importantly, anti-lexicalism allows us to focus on the single capacity of Merge in order to discuss the origins of syntax and the lexicon of human language. Lexicalism requires us to consider the two generative modules separately. Thus anti-lexicalism offers a superior perspective in evolutionary terms, too, even though its practitioners do not generally take the evolutionary issues into consideration.

**Fig. 4:**
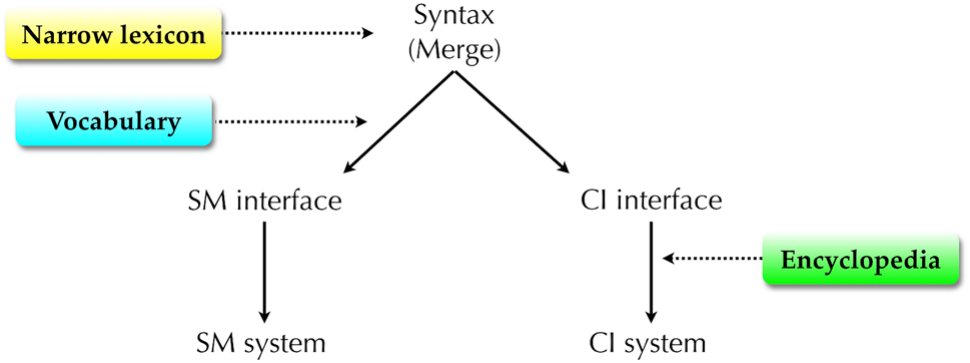
The structure of grammar in Distributed Morphology (adapted from Fujita 2017; cf. Embick and Noyer 2007; Marantz 1997). The elements of the narrow lexicon are used by syntax (Merge) to generate hierarchical structure. On the morphophonological side, this structure is mapped to the SM system via the SM interface (PF) based on the information of the vocabulary. On the conceptual-semantic side, it is mapped to the CI system via the CI interface (LF) by referring to the encyclopedia

Distributed Morphology (DM; Halle and Marantz 1993 et seq.) is the most widely accepted framework of anti-lexicalism. DM rejects the traditional notion of the lexicon and argues that the elements of the lexicon are distributed over three non-generative lists: the narrow lexicon, the vocabulary, and the encyclopedia. The narrow lexicon is a list of lexical items in the sense of “the atoms of computation” (Berwick and Chomsky 2016: 66) to which Merge applies. The vocabulary is a list containing pairs of phonological exponents and their conditions for realization. This list is referred to when syntactic structure is mapped to the SM interface and the SM system. The encyclopedia is a list of idiosyncratic meanings of each lexical item or idiom-like expression and is consulted in the semantic interpretation by the CI system via the CI interface. Whereas in the traditional view of grammar all of syntactic, phonological, and semantic properties of words belong to the single lexicon that feeds syntax, DM maintains that these properties are separately listed and used in distinct grammatical processes. Figure 4 illustrates this structure of grammar (see also Fig. 1).

In addition to being a model that embodies anti-lexicalism, DM can systematically explain syntactic-morphological phenomena by distributing the multiple elements that were once attributed to words. This distributed model is also useful in discussing the evolution of language. To put it concretely, by decomposing the complex entity of “words” unique to human language into smaller and more primitive elements as atoms of computation, phonological information, and conventional meanings, DM makes it easier to examine such evolutionary issues as what the precursors of these elements are, whether they are culturally or biologically evolved, or how they relate to the abilities found in other species.

As stated above, the hierarchical structure generated by Merge and its mapping to the CI interface (internalization) is universal but its phonological counterpart and conventional meaning vary across individual languages. Then the vocabulary and the encyclopedia are socioculturally formed entities and only the narrow lexicon counts as a biologically evolved capacity of the human species. Thus, we discuss the evolution of primitive syntactic objects (lexical items) composing the narrow lexicon in the next section.

## The evolution of roots and functional morphemes

In DM, lexical items are divided into two types: roots and functional morphemes. In the traditional terms of generative grammar, the former correspond to such lexical categories as verbs, nouns, adjectives/adverbs, and possibly prepositions, the latter to functional categories that contain everything else. We discuss the evolution of each.

### Roots

Roots are conceptual elements that form an open class. Roots are assumed to be category-free and specified as a particular part of speech when they are combined with categorizers.^4^ There is still no consensus on the other properties of roots, especially on what kind of information (syntactic, phonological, or semantic) roots contain. Regardless of the particular assumption one may adopt, however, it is necessary to consider the continuity between human language and cognitive capacities found in other animals to understand the biological origin of roots.

Roots/lexical items are often regarded as primitive concepts in the literature (Berwick and Chomsky 2016; Chomsky 2005, 2007, 2009). The long-standing dominant view in theoretical linguistics is that concepts are unique to humans with language. However, studies of animal behavior have shown that nonhuman species also have concepts like ours (for just a few examples, see Schlenker et al. (2016b), Seyfarth et al. (1980), Seyfarth and Cheney (2012), etc. for conceptual meanings of monkey calls, and Bugnyar (2007), Pepperberg (1987, 2002), and Wright and Cumming (1971), for conceptual abilities of birds; see also Fitch (2010) for an extensive discussion). Therefore, it is reasonable to suppose that roots as concepts are shared with other species to some extent.

Are concepts in other species exactly the same as roots/concepts of human language? While admitting that other species also have various conceptual structures, Berwick and Chomsky (2016) claim that they crucially differ from human linguistic concepts in that only the latter are “mind-dependent” in the sense that they do not refer directly (mind-independently) to objects or events in the outside world. This observation may be correct as long as animal communication is concerned, but since, as Chomsky himself has emphasized for many decades, language is primarily a mental computational system for internal cognitive functions rather than a mere tool for communication, what we really have to figure out is the nature of concepts in other species used in the process of internal cognition. Given that they retain some episodic memory and solve complicated tasks requiring reasoning and planning, it is not so clear that their concepts are indeed mind-independent. Further progress in comparative cognitive psychology will settle this issue.

Another point to make is that the richness of concepts in human language may be largely attributed to the mechanism of Merge recursively and infinitely combining simple concepts into more complex, structured ones, rather than the complexity of individual concepts. In addition, as noted above, externalization of concepts is also likely to be responsible for the richness of human concepts because it not only facilitates the manipulation of concepts by providing them with sound or visual information which will improve their tractability, but also contributes to the elaboration and expansion of conceptual meanings by enabling humans to share and inter-subjectively define them (Fujita 2020). This leads to further generation of hierarchical structure composed of concepts containing pragmatic and phonological meaning (see also Chomsky et al. (2019) for relevance of externalization on conceptual meanings).

It may be premature, then, to conclude that the mind-dependent nature and richness of human concepts/lexical items are indeed unique to the human conceptual system. This conclusion could end up overemphasizing the peculiarity of human concepts and dismissing the evolution of lexical items as a mystery, as in Berwick and Chomsky (2016). It seems more reasonable to think that the difference between human and animal concepts is a reflection of whether Merge can operate on them, instead of sheer evolutionary discontinuity between them. This line of thinking makes it possible to reduce the crucial change that led to the emergence of human language to the evolution of syntactic computation alone, much in line with the minimalist approach that aims to minimize the language-specific components of the human language faculty (Hauser et al.’s (2002) FLN). Needless to say, that this Merge is innate and part of UG, to be explained in terms of human evolution, is another story. We have suggested otherwise above, the implication being that neither Merge nor lexical items belong to UG.

### Functional morphemes

Functional morphemes are bundles of grammatical features such as tense, number, and person, without phonological specification. All elements other than roots/lexical categories are conventionally classified as functional morphemes. However, given the range of their functions and the diversity across languages, it is difficult to discuss their evolutionary origin as long as we simply put them together as miscellaneous elements other than lexical categories. We propose instead that functional morphemes are classified into two types: contextual functional categories (CFCs) and structural functional categories (SFCs).

CFCs play an essential role in constructing propositional content of linguistic structure by representing the spatiotemporal information. These include morphemes such as tense, aspect, and demonstrative. In contrast, SFCs are elements such as complementizer, case marker, and agreement marker indicating the structural relationship between constituents, which can be obscured by linearization. This subclassification of functional morphemes based on their functions makes it easier to explore their respective evolutionary backgrounds.

CFCs are adaptive in that they enhance the creativity of linguistic expressions. The separation of referential function from lexical categories as CFCs makes it possible to freely combine lexical contents with reference markers to create various expressions that are not limited to specific contexts or here-and-now interpretation, such as *this cat* or *that cat*; *the rats you will see in your dream* or *the rats you saw in your dream*. The use of the nouns themselves in these phrases is neither referential in itself nor is limited to the here-and-now interpretation depending on what other elements cooccur, and this yields the property of displacement in the sense of Hockett (1960) (not in the sense of movement described earlier). Although nonhuman species may also have concepts detached from here-and-now, they lack the means to express or externalize them. The emergence of CFCs, which enabled humans to express displacement, must be a turning point in the advanced expressive power of language and linguistic thought. It is unlikely that CFCs emerged independently of lexical categories because they are equally important in expressing semantic content. Thus we propose below that the emergence of CFCs is attributed to the separation of referential and lexical contents from proto-lexical categories which did not differentiate them.

In comparison with CFCs, SFCs do not bear much conceptual meaning composing propositional contents and their main function is to mark structural information. They are more relevant to externalization than internalization. Although structural relations within a linguistic expression may be obvious to the one who produces it, they can get obscured in the process of linearization for externalization, as already illustrated by the kind of structural ambiguity as in (1)–(3) above.

As stated above, generative grammar holds that all cross-linguistic diversities boil down to externalization while internalization is a universal process, and this implies that SFCs as elements of external forms are one source of such diversities, and also that SFCs do not belong to the narrow lexicon. As well attested, different languages employ different means to indicate structure-dependent information, such as morphemes of SFCs, word order, and prosody. For example, Japanese resolves the structural ambiguity of (13), which has the two interpretations of (14a, b), by prosody and context, while in (15) French does so by an agreement marker (an SFC). Compare:

(13)

(14)
(15)
(16)

(In (16) the preposition *du* is omitted.) In (15a)–(16a), the adjective *blanc* agrees with the masculine *livre*, which clearly shows that it is the book that is white. In (15b)–(16b), *blanche* agrees with the feminine *jaquette*, indicating that the jacket is white.

**Fig. 5:**
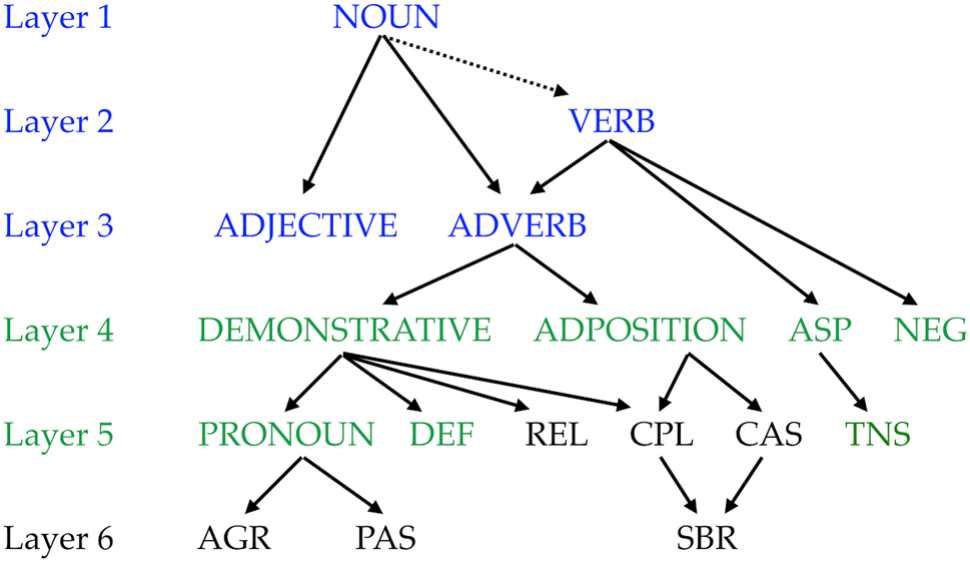
Layers of grammatical development (adapted from Heine and Kuteva 2007; see also Heine and Kuteva 2012). AGR stands for agreement marker, ASP for (verbal) aspect, CAS for case marker, CPL for complementizer, DEF for marker of definiteness (definite article), NEG for negation marker, PAS for passive marker, REL for relative clause marker, SBR for subordinating marker of adverbial clauses, TNS for tense marker. The dotted arrow indicates an indirect relation

Given the discussion so far, the evolutionary background of SFCs seems to be deeply rooted in the development of externalization for communication. Hence, we consider that SFCs emerged later in different cultures and languages when externalization of hierarchical structure became necessary (the cultural evolution of language). A probable evolutionary factor that led to the emergence of SFCs is “grammaticalization.” Grammaticalization refers to the process in which morphemes (or words) with concrete meanings come to express more abstract meanings and grammatical roles (semantic bleaching). It is often observed that lexical categories or CFCs are diverted into SFCs. For example, the English demonstrative *that* has come to be used as a complementizer; and the French pronoun *il*, which derived from the Latin demonstrative *ille*, functions as an agreement marker in nonstandard French. Based on the analysis of grammaticalization across various languages, Heine and Kuteva (2007, 2012) propose the six-layered model of the development of grammatical categories as in Fig. 5, where each layer has developed on the basis of the previous layer (Heine and Kuteva 2012).

This model has great significance not only for cultural evolution but for biological evolution of language. We can apply it to our discussion so far and say that the elements of layers 1, 2, and 3 correspond to lexical categories (indicated in blue letters), that layer 4 and part of layer 5 correspond to CFCs (indicated in green), and that the other parts of layer 5 and the entire layer 6 are SFCs. Thus, CFCs emerged from lexical categories, and lexical categories and CFCs underlie the development of SFCs.

## Disintegration of lexical and functional categories

The previous section discussed the evolutionary issues of roots and functional morphemes separately on the premise that elements of the narrow lexicon can be divided into these two types. However, it is necessary first to explain why the human lexicon contains these two categories, lexical and functional. This section reviews and compares two promising (and contrasting) hypotheses concerning this point, namely, the Integration Hypothesis and the Disintegration Hypothesis.

### Integration hypothesis

The Integration Hypothesis (IH) is proposed by Miyagawa et al. (2013), Miyagawa et al. (2014), and Miyagawa (2017) (see also Nóbrega and Miyagawa 2015). IH maintains that human language with infinite hierarchical structure emerged from the integration of the two pre-adapted systems, the expressive system (E system) of songbirds and the lexical system (L system) of primate calls. The E system performs the formal or “expressive” function without forming meaning units, while the L system describes predicates or objects by assembling several elements into meaningful units. In the context of generative grammar, the E system corresponds to functional categories and the L system to lexical categories.

Although each of these two systems has only a finite-state grammar, IH argues that their integration results in a nonfinite state grammar in human language. Miyagawa et al. (2013) and Miyagawa et al. (2014) analyze the structure of human language as the alternate combination of the E system (E layer) and the L system (L layer), as shown in (17). This structure is expressed by a recursive and infinite rule as in (18). IH derives the recursive structure of human language from such combinations of the two systems.

(17)

(18) Rules of alternate structure (Miyagawa et al. 2013: 4)
  (i) EP → E LP
  (ii) LP → L EP

As to how the integration of the two systems happened, Miyagawa et al. (2014) attribute it to the nonfinite state processes of movement and agreement which link the two layers to each other, based on the observation that movement typically occurs from the L layer to the E layer. On the other hand, Nóbrega and Miyagawa (2015) suggest that the integration in question is caused by the emergence of full-fledged Merge.

IH is an interesting idea in that: (1) it presents a natural scenario of language evolution from the perspective of biological evolution where pre-existing traits underlie a new trait, and (2) it promotes intensive discussion on the evolutionary continuity of human language and animal communication.

Nonetheless, there are two significant problems with IH. The first problem is that the E system of birds and the L system of nonhuman primates cannot be equated with functional and lexical categories of human language. The function of the E system, which conveys a single message with an entire sound sequence, is quite different from that of the functional categories of human language that define propositional contents with spatiotemporal information or indicate structural relation. Besides, as mentioned above, the L system in other animals’ communication directly refers to the external world, unlike mind-dependent human language. In addition, the distinction between the L and E systems in other species remains vague. The L system of primate alarm calls, for instance, also functions as an E system because it not only describes a situation where a predator is approaching but it also conveys fear, alertness and a command to escape.

The second problem concerns the circular or redundant nature of the suggested explanation of the integration in question. As stated above, IH maintains that the integration took place as a result of movement/agreement or Merge, but if language already had these devices, there is no need to derive the recursive nature of linguistic structure from the integration itself. The effect of IH can be safely discarded in a Merge-based explanation of language evolution. Turning back to the issue of why human language has lexical and functional categories, IH maintains that they each were already present in pre-existing animal communication systems, to be later integrated in human language. We have pointed out some serious problems with this scenario.

### Disintegration hypothesis

We believe it is more reasonable to think that the E system and the L system are undifferentiated in animal communication, only to be finally differentiated in human language as lexical categories and functional categories, respectively. We call this hypothesis the Disintegration Hypothesis (DH: Fujita and Fujita 2016).

Bronowski (1977) already pointed out that animal communication differs from human language in that the former does not (or cannot) separate referential content and emotional charge, and DH is a natural extension of this observation. Human language has a division of labor between lexical and functional categories, the latter expressing specific information related to actual circumstances.^5^ This division made it possible for human language to create flexible expressions not bound by a limited set of referents and situations. This release from the immediate external stimuli is one crucial factor of the mind-dependent nature of human lexical items. The emergence of CFCs can now be explained by the disintegration of the E system and the L system. But then how did this disintegration take place?

Many researchers assume the existence of some form of protolanguage long before the advent of human language.^6^ We assume that the disintegration occurred in the transition from protolanguage to human language, not from animal communication to human language.^7^ We further speculate that this disintegration was promoted by a mutual segmentation of context and signal, along the lines of Okanoya’s (2007) and Okanoya and Merker’s (2007) String-Context Mutual Segmentation Hypothesis. The general idea is described as follows. Suppose our ancestors had string A′ for context A, and B′ for B. Suppose also that A and B share a common sub-context C, and that A′ and B′ share a common sub-string C′. Such a situation will gradually allow the establishment of string C′ for context C. In accordance with this hypothesis, we propose that further mutual segmentation will take place, allowing a common sub-string shared by C′ and another string D′, say E′, to bear some common referential information E. Such a series of mutual segmentation will cause the disintegration of the L system and the E system.

Furthermore, along with this multiple mutual segmentation, the cognitive abilities necessary for concept formation, such as schematization and categorization that analyze and classify abstract similarity or structure, must have played an equally important role in the disintegration process. When these cognitive abilities worked on symbols in protolanguage, whose referential functions and conceptual content we assume were still undifferentiated, it became possible to extract abstract commonality including spatiotemporal distance and mental attitudes from these symbols. See Hoshi (2018, 2019) for possible relations between DH and categorization.

Suppose protolanguage had expressions like (19a, b), in which the speaker reports on certain events she actually saw in the past. Uppercase notation means that both referential functions and conceptual content are included in one “proto-word.” If our ancestors at that time were already able to segment each element of (19), they would easily see that these expressions were all composed of the same two types of concept, things and actions. They would also see that these expressions all indicate specific things (definiteness) and specific actions which already happened (past tense). The former property is categorized by the, the latter by past, giving rise to (20a, b) and (21a, b), respectively. In human language, both of these properties are denoted by distinct categories, as in (22).

(19) a. APPLE FALL.
  b. STONE ROLL.
(20) a. The apple Fall
  b. The Stone ROLL.
(21) a. APPLE fall-past
  b. STONE roll-past
(22) a. The apple fell
  b. The stone rolled

Comparative studies show that other species also display category recognition (Wright and Cunning 1971 for the classic example) and even a kind of conceptual metaphor (Dahl and Adachi 2013). It is likely that the elaboration of concepts based on these cognitive abilities not only promoted the segmentation of proto-words but were also involved in their formation.

Needless to say, all the discussions so far are yet to be subjected to empirical verification via intensive studies on human evolution, animal communication/cognition, neuroscience of language, etc. Nevertheless, we contend that our hypothesis merits a serious consideration in that it provides a firm theoretical foothold for studying human language evolution in light of its continuity with animal communication/cognition.

## Concluding remarks

To achieve a natural understanding of human language evolution, in this article we have presented our hypotheses on how hierarchical syntax and the lexicon—the two modules of language which have been believed to be truly uniquely human—may have evolved from pre-existing precursors. Specifically, we have argued that the fundamental computational operation Merge derived from motor action planning, and that lexical and functional categories derived from animal cognition and a presumed proto-lexicon of proto-language via the process of disintegration. We have built our arguments on recent developments in generative grammar but departed from its core idea in some crucial respects. Most importantly, we maintain that every component of language has evolutionary continuity with other species’ cognitive capacities; there is no FLN in the sense of Hauser et al. (2002). We also believe that language evolved in a gradual manner, not as abruptly as commonly claimed in generative grammar. We hope our hypotheses will offer a new perspective on language evolution which is in good harmony with the general picture of biological evolution.

## Footnotes

1 For some comprehensive discussions on recent findings in animal cognition and communication, see Andrews (2015), Olmstead and Kuhlmeier (2015), Wynne and Udell (2013), etc. See also Fitch (2019).

2 Technically, Merge comes in two kinds, set-Merge and pair-Merge. In this article we only focus on set-Merge. The evolutionary status of pair-Merge, or whether pair-Merge really exists, is unclear to us, and we exclude pair-Merge from our discussion. See Chomsky (2004) for how set-Merge and pair-Merge differ from each other.

3 This is not the only way to explore the evolutionary origins of Merge. Hoshi (2018, 2019) argues that Merge may have evolved from the (set-formation part of) categorization, where categorization can be thought of as a combination of set-formation and labeling. We assume that these two hypotheses are not mutually exclusive but both can be part of a larger picture, though we will not discuss the topic here.

4 DM claims that categorizers are a kind of functional morpheme listed in the narrow lexicon. As such, it is rather difficult to explore their evolutionary origin because they presume language-specific properties which are an end product of language evolution. It may be the case that categorizers are just a theoretical construct not to be listed in the narrow lexicon as real objects. See Fujita (2017b) for some discussion.

5 Abstraction of the L system and patterning of the E system mentioned in Miyagawa (2017) seems to be related to this feature of language although he considers these features as a cause of the integration of the E and L systems, not the result of their disintegration.

6 See Bickerton (1990) for the idea of protolanguage. See also Arbib and Bickerton (2010) for different views on what protolanguage was like. The relevance of protolanguage has been denied in the generative tradition but it is mostly only in the specific sense that Merge has no evolutionary precursor. We have already objected to this view above.

7 The crucial difference between protolanguage and animal “language” we take to be that protolanguage was already adaptive for both thought and communication, whereas animal language is for communication alone. Many agree that *Homo erectus* about 1.5 million years ago already had some form of protolanguage, but the relation between protolanguage and human language is not always clear. It can be argued that protolanguage evolved into, or replaced by, human language, or that they are two distinct, potentially coexisting systems. The observation that linear syntax is operative even today in addition to hierarchical syntax suggests that the coexistence scenario is more likely.

## References

[CR1] Andrews K (2015). The animal mind: an introduction to the philosophy of animal cognition.

[CR2] Arbib MA, Bickerton D (2010). The emergence of protolanguage: holophrasis vs compositionality.

[CR3] Baker M (1985). The mirror principle and morphosyntactic explanation. Linguist Inq.

[CR4] Benítez-Burraco A, Kempe V (2018). The emergence of modern languages: has human self-domestication optimized language transmission?. Front Psychol.

[CR5] Berwick RC, Di Sciullo AM, Boeckx C (2011). All you need is Merge: biology, computation and language from the bottom up. The biolinguistic enterprise: new perspectives on the evolution and nature of the human language faculty.

[CR6] Berwick RC, Chomsky N (2016). Why only us: language and evolution.

[CR7] Bickerton D (1990). Language and species.

[CR8] Boeckx C (2008). Bare syntax.

[CR9] Boeckx C, Grohmann KK (2013). The Cambridge handbooks of biolinguistics.

[CR10] Bronowski J (1977). A sense of the future: essays in natural philosophy.

[CR11] Bugnyar T (2007). An integrative approach to the study of ‘theory-of-mind’-like abilities in ravens. Jpn J Anim Psychol.

[CR12] Chomsky N (1965). Aspects of the theory of syntax.

[CR13] Chomsky N (1986). Knowledge of language: its nature, origin, and use.

[CR14] Chomsky N (1995). The minimalist program.

[CR15] Chomsky N, Belletti A (2004). Beyond explanatory adequacy. Structures and beyond: the cartography of syntactic structures.

[CR16] Chomsky N (2005). Three factors in language design. Linguist Inq.

[CR17] Chomsky N (2007). Of minds and language. Biolinguistics.

[CR18] Chomsky N (2009). Cartesian Linguistics: a chapter in the history of rationalist thought.

[CR19] Chomsky N, Gallego ÁJ, Ott D (2019). Generative grammar and the faculty of language: insights, questions, and challenges. Catalan J Linguist.

[CR20] Conway CM, Christiansen MH (2001). Sequential learning in non-human primates. Trends Cogn Sci.

[CR21] Dahl CD, Adachi I (2013). Conceptual metaphorical mapping in chimpanzees (Pantroglodytes). eLife.

[CR22] Embick D, Noyer R, Ramchand G, Reiss C (2007). Distributed morphology and the syntax-morphology interface. The Oxford handbook of linguistic interfaces.

[CR23] Everaert MBH, Huybregts MAC, Chomsky N, Berwick RC, Bolhuis JJ (2015). Structures, not strings: linguistics as part of the cognitive sciences. Trends Cogn Sci.

[CR24] Fitch WT (2010). The evolution of language.

[CR25] Fitch WT (2019). Animal cognition and the evolution of human language: why we cannot focus solely on communication. Phil Trans R Soc B.

[CR26] Fujita K (2009). A prospect for evolutionary adequacy: merge and the evolution and development of human language. Biolinguistics.

[CR27] Fujita K, Roeper T, Speas M (2014). Recursive Merge and human language evolution. Recursion: complexity in cognition.

[CR28] Fujita K, Fujita K, Boeckx C (2016). On certain fallacies in evolutionary linguistics and how one can eliminate them. Advances in biolinguistics: the human language faculty and its biological basis.

[CR29] Fujita K (2017). On the parallel evolution of syntax and lexicon: a merge-only view. J Neurolinguistics.

[CR30] Fujita H (2017b) On the emergence of human language: with special reference to the evolution of lexical items. Master’s Dissertation, Kyoto University

[CR31] Fujita H (2020). Co-evolution of internalization and externalization in the emergence of the human lexicon: a perspective from generative grammar and cognitive linguistics. Evol Linguist Theory.

[CR32] Fujita K, Fujita H (2016) Integration or disintegration? In: Roberts SG, Cuskley C, McCrohon L, Barceló-Coblijn L, Fehér O, Verhoef T (eds) The evolution of language: Proceedings of the 11th international conference (EVOLANG11), pp 430–432. http://evolang.org/neworleans/papers/16.html

[CR33] Greenfield PM (1991). Language, tools, and brain: the ontogeny and phylogeny of hierarchically organized sequential behavior. Behav Brain Sci.

[CR34] Greenfield PM (1998). Language, tools, and brain revisited. Behav Brain Sci.

[CR35] Halle M, Marantz A, Hale K, Keyser SJ (1993). Distributed morphology and the pieces of inflection. The view from building 20: essays in linguistics in honor of Sylvain Bromberger.

[CR36] Hauser MD (2009). Origin of the mind. Sci Am Sept.

[CR37] Hauser MD, Watumull J (2017). The universal generative faculty: the source of our expressive power in language, mathematics, morality, and music. J Neurolinguistics.

[CR38] Hauser MD, Chomsky N, Fitch WT (2002). The faculty of language: what is it, who has it, and how did it evolve?. Science.

[CR39] Hayashi M, Takeshita H (2020). Object sorting into a two-dimensional array in humans and chimpanzees. Primates.

[CR40] Heine B, Kuteva T (2007). The genesis of grammar: a reconstruction.

[CR41] Heine B, Kuteva T, Tallerman M, Gibson KR (2012). Grammaticalization theory as a tool for reconstructing language evolution. The Oxford handbook of language evolution.

[CR42] Hockett C (1960). The origin of speech. Sci Am.

[CR43] Hoshi K (2018). Merge and labeling as descent with modification of categorization: a neo-Lennebergian approach. Biolinguistics.

[CR44] Hoshi K (2019). More on the relations among categorization, Merge and labeling, and their nature. Biolinguistics.

[CR45] Jackendoff R, Wittenberg E (2016). Linear grammar as a possible stepping-stone in the evolution of language. Psychon Bull Rev.

[CR46] Kayne RS (1994). The antisymmetry of syntax.

[CR47] Marantz A (1997) No escape from syntax: don’t try morphological analysis in the privacy of your own lexicon. Univ Pa Working Pap Linguist 4(2): 201–225

[CR48] Marcus GF (2006). Cognitive architecture and descent with modification. Cognition.

[CR49] Martins PT, Boeckx C (2016). What we talk about when we talk about biolinguistics. Linguist Vanguard.

[CR50] Matsuzawa T, McGrew WC, Marchant LF, Nishida T (1996). Chimpanzee intelligence in nature and in captivity: isomorphism of symbol use and tool use. Great ape societies.

[CR51] Miyagawa S, Watanabe S, Hofman M, Shimizu T (2017). Integration hypothesis: a parallel model of language development in evolution. Evolution of the brain, cognition, and emotion in vertebrates.

[CR52] Miyagawa S, Clarke E (2019). Systems underlying human and Old World Monkey communication: one, two or infinite. Front Psychol.

[CR53] Miyagawa S, Berwick RC, Okanoya K (2013). The emergence of hierarchical structure in human language. Front Psychol.

[CR54] Miyagawa S, Ojima S, Berwick RC, Okanoya K (2014). The integration hypothesis of human language evolution and the nature of contemporary languages. Front Psychol.

[CR55] Nóbrega VA, Miyagawa S (2015). The precedence of syntax in the rapid emergence of human language in evolution as defined by the integration hypothesis. Front Psychol.

[CR56] Okanoya K (2007). Language evolution and an emergent property. Curr Opin Neurobiol.

[CR57] Okanoya K, Merker B, Nehaniv CL, Cangelosi A, Lyon C (2007). Neural substrates for string-context mutual segmentation: a path to human language. Emergence of communication and language.

[CR58] Olmstead MC, Kuhlmeier VA (2015). Comparative cognition.

[CR59] Pepperberg IM (1987). Acquisition of the same/different concept by an African Grey parrot (*Psittacus erithacus*): learning with respect to categories of color, shape, and material. Anim Learn Behav.

[CR60] Pepperberg IM (2002). Cognitive and communicative abilities of Grey Parrots. Curr Dir Psychol Sci.

[CR61] Progovac L (2015). Evolutionary syntax.

[CR62] Schlenker P, Chemla E, Arnold K, Zuberbühler K (2016). *Pyow-hack* revisited: two analyses of putty-nosed monkey alarm calls. Lingua.

[CR63] Schlenker P, Chemla E, Zuberbühler K (2016). What do monkey calls mean?. Trends Cogn Sci.

[CR64] Seyfarth RM, Cheney DL, Gibson K, Tallerman M (2012). Primate social cognition as a precursor to language. Oxford handbook of language evolution.

[CR65] Seyfarth RM, Cheney DL, Marler P (1980). Vervet monkey alarm calls: semantic communication in a free-ranging primate. Anim Behav.

[CR66] Suzuki TN, Wheatcroft D, Griesser M (2018). Call combinations in birds and the evolution of compositional syntax. PLoS Biol.

[CR67] Thomas J, Kirby S (2018). Self domestication and the evolution of language. Biol Philos.

[CR68] Wilkins AS, Wrangham RW, Fitch WT (2014). The ‘domestication syndrome’ in mammals: a unified explanation based on neural crest cell behavior and genetics. Genetics.

[CR69] Wright AA, Cumming WW (1971). Color-naming functions for the pigeon. J Exp Anal Behav.

[CR70] Wynne CDL, Udell MAR (2013). Animal cognition: evolution, behavior and cognition.

